# Peptidyl Fluoromethyl Ketones and Their Applications in Medicinal Chemistry

**DOI:** 10.3390/molecules25174031

**Published:** 2020-09-03

**Authors:** Andrea Citarella, Nicola Micale

**Affiliations:** Department of Chemical, Biological, Pharmaceutical and Environmental Sciences, University of Messina, Viale Ferdinando Stagno D’Alcontres 31, I-98166 Messina, Italy; acitarella@unime.it

**Keywords:** peptidyl fluoromethyl ketones, fluorinated peptides, cysteine proteases, serine proteases, enzymatic inhibitors, cathepsin, caspase, SARS-CoV M^pro^

## Abstract

Peptidyl fluoromethyl ketones occupy a pivotal role in the current scenario of synthetic chemistry, thanks to their numerous applications as inhibitors of hydrolytic enzymes. The insertion of one or more fluorine atoms adjacent to a *C*-terminal ketone moiety greatly modifies the physicochemical properties of the overall substrate, especially by increasing the reactivity of this functionalized carbonyl group toward nucleophiles. The main application of these peptidyl α-fluorinated ketones in medicinal chemistry relies in their ability to strongly and selectively inhibit serine and cysteine proteases. These compounds can be used as probes to study the proteolytic activity of the aforementioned proteases and to elucidate their role in the insurgence and progress on several diseases. Likewise, if the fluorinated methyl ketone moiety is suitably connected to a peptidic backbone, it may confer to the resulting structure an excellent substrate peculiarity and the possibility of being recognized by a specific subclass of human or pathogenic proteases. Therefore, peptidyl fluoromethyl ketones are also currently highly exploited for the target-based design of compounds for the treatment of topical diseases such as various types of cancer and viral infections.

## 1. Introduction

**Figure 1:**
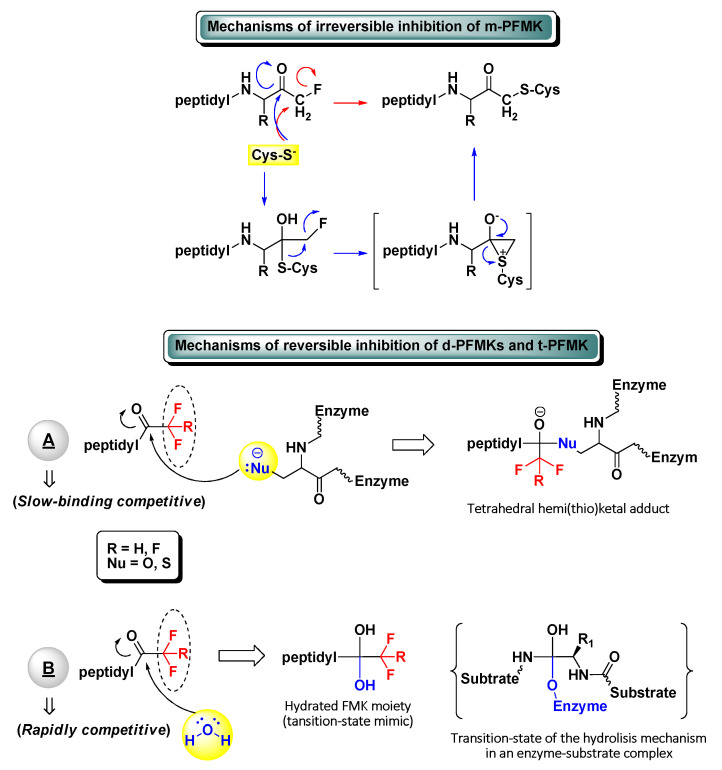
Schematic representation of the possible inhibitory mechanisms of action of all types of peptidyl fluoromethyl ketones (PFMKs).

The development of peptidyl fluoromethyl ketones (PFMKs) has received over the last few decades a growing interest in drug discovery, since these types of compounds may be employed as substrates for a wide variety of biological targets [1]. The fluorinated electrophilic moieties of such substrates offer several advantages in comparison to other equivalent chemical units in terms of reactivity, selectivity, and therapeutic relevance. The mono-fluorinated function group, for instance, shows in general high reactivity and selectivity for cysteine proteases. At the same time, it exerts poor irreversible inhibition towards serine proteases and does not show any significant reactivity towards bionucleophiles such as glutathione [2]. On the other hand, a peptidic framework often represents the most foreseeable recognition motif for new biological targets and then the starting point of the target-based drug design. Usually, excellent substrate specificity can be fairly easily achieved by varying a single amino acid of the peptide sequence, with the position next to the electrophilic moiety (P_1_) being largely the most sensitive. As a matter of fact, various peptides bearing a *C*-terminal mono-fluoromethyl ketone (m-FMK) warhead have been used as selective activity-based probes for important druggable enzymatic targets, e.g., cathepsins (Cats), caspases (Casps), calpain I, SENPs, and *N*-glycanase. Unfortunately, their development as drugs has been compromised by the in vivo metabolic conversion of the m-FMK moiety into the highly toxic fluoroacetate [3]. In regard to the kinetics and mechanism of action of PFMKs, the high stability of the C–F bond was expected to lead a slow-binding reversible competitive inhibition of these nucleophilic targets (mostly hydrolytic enzymes) by electrophiles forming hemi(thio)ketal adducts (Figure 1). However, this has been ascertained for the majority of the tri-fluorinated derivatives (i.e., t-PFMKs) and very often observed in di-fluorinated derivatives (i.e., d-PFMKs), whereas it is well known that mono-fluorinated derivatives (i.e., m-PFMKs) are usually irreversible inhibitors leading to the formation of a covalent thioether adduct. The latter may be formed through the direct SN2 displacement of the fluoride group by the cysteine thiolate group or, more likely, through a two-step mechanism involving a thioemiketal and a three-membered sulfonium intermediate that instantly rearranges to afford the eventual thioether adduct (Figure 1) [4]. d-PFMKs and t-PFMKs, instead, have found a wide range of applications in synthetic medicinal chemistry, especially as enzyme inhibitors. The presence of additional fluorine atoms within their *C*-terminal warhead increases the electrophilicity of the FMK carbonyl group and makes them more susceptible to undergoing nucleophilic attack, also by the catalytic –OH group of serine proteases. Additionally, at variance with m-PFMKs, d-PFMKs and t-PFMKs do not present significant drawbacks related to the metabolism of the FMK moiety. These two fluorinated moieties exhibit another important characteristic; they can easily hydrate, depending on the nature of the amino acids they are bound to, forming very stable *gem*-diols which mimic the tetrahedral adduct of the transition-state of the enzyme-substrate hydrolysis mechanism (Figure 1). Therefore, the resulting PFMKs may also act exclusively as transition-state competitive analogues leading to rapidly reversible competitive inhibition [5,6]. In most cases, a mixture of the two FMK forms (i.e., carbonyl and hydrated) in different ratio is observed in solution, and a dual inhibition mechanism of the PFMKs is detected consequently.

This review gives an thorough overview of the PFMKs reported in literature so far together with the biological activity, underlining their importance in the study of biochemical pathways and molecular mechanisms that regulate the development of relevant human pathologies. Furthermore, the review covers recent advances in drug discovery for these types of compounds, whose target-based design is becoming notable in view of their ability to selectively inhibit specific hydrolytic enzymes.

## 2. Peptidyl Mono-Fluoromethyl Ketones (m-PFMKs)

### 2.1. Cathepsins Inhibitors

**Figure 2:**
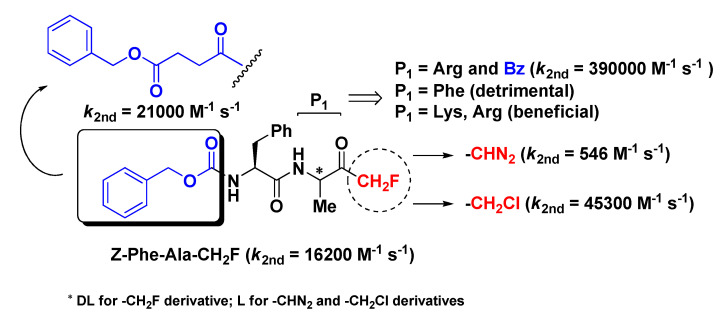
Chemical structure, Cat-B inhibitory potency, and most relevant modifications of the first synthesized m-PFMK.

m-PFMKs were the first to be synthesized and reported in the literature as potent and irreversible inhibitors of human cathepsin B (Cat-B) by David Rasnick in a comparative study with the diazomethyl and chloromethyl ketone analogues [7]. Cat-B is a lysosomal cysteine protease of the papain-like family that plays a leading role in cellular proteolysis, apoptotic processes, and development/proliferation of T lymphocytes. In particular, up-regulation of Cat-B is implicated in the genesis of various pathological conditions such as tumors and autoimmune diseases [8]. In this study, it emerged that the dipeptide derivative Z-Phe-Ala-CH_2_F was a good inhibitor of the intended enzymatic target with a second-order rate constant value of 16,200 M^−1^ s^−1^ (Figure 2), much higher (~30-fold) than that of the diazomethyl analogue (546 M^−1^ s^−1^) and lower than that of the chloromethyl analogue (45,300 M^−1^ s^−1^), whose relatively strong reactivity as electrophile may lead to indiscriminate alkylation of non-target molecules in vivo and appearance-related side effects. This m-PFMK was synthesized by a modified Dakin–West reaction, which leads to complete racemization of the model peptide (i.e., P_1_ site), at variance with the other two parent peptides which were obtained in optically pure form. Rasnick and co-workers’ investigations on Z-Phe-Ala-CH_2_F continued by means of in vivo models of arthritic rat knee joints, wherein both intravenous and oral administration of this m-PFMK enabled them to demonstrate that significant reduction of inflammation and cartilage damage are related to high Cat-B activity [9]. The importance of the stereochemistry at the P_1_ site was noted by Esser R.E. et al. in the early 1990s, by obtaining the two diastereoisomers Z-l-Phe-l-Val-CH_2_F and Z-l-Phe-d-Val-CH_2_F and proving that the m-PFMK composed of natural amino acids was > 100-fold more active than the other in vitro on purified human Cat-B and, in parallel, that only Z-l-Phe-l-Val-CH_2_F was able to significantly reduce in vivo the severity of the arthritis clinical signs in the above-mentioned murine model of adjuvant-induced arthritis after oral administration [10].

Yagel S. et al. indirectly proved that Z-Phe-Ala-CH_2_F is also an effective inhibitor of Cat-L, another lysosomal cysteine protease of papain-like family that plays a major role in metastatic processes [11]. The m-PFMK was able to significantly reduce the invasion of human amnion by two types of murine cancer cells, i.e., B16BL6 melanoma and C3-L1 mammary adenocarcinoma cells, which are known to be good producers of Cat-L [12].

Soon after Rasnick’s pioneer study, Shaw’s group carried out a similar comparative analysis on m-PFMKs [13]. By switching the P_1_ amino acid residue (Ala) of the above-depicted lead structure with a Phe residue, they obtained a Cat-B inhibitor compound one order of magnitude less potent than the reference m-PFMK, proving that this modification does not improve the specificity toward this target (Figure 2). They also performed kinetics studies (affinity labeling technique), which confirmed the irreversible mechanism of inhibition of this type of fluorinated derivatives and their lower alkylation rate of the enzyme active site compared to the chlorinated analogues. These m-PFMKs were obtained by conversion of phthaloyl amino acids into their fluoromethane derivatives, a method that still leads to a P_1_ residue in racemic form. Shaw’s group continued its investigation on this class of compounds by introducing positively charged amino acid residues (such as Lys and Arg) at the P_1_ position and by continuing to test these m-PFMKs against Cat-B, a bacterial cysteine protease that specifically cleaves on the COOH-side of Arg residue (i.e., clostripain) and a panel of serine proteases homologous with trypsin [14,15]. From these studies clearly emerged that this type of modification at the P_1_ site outstandingly affected the inhibitory potency of the m-PFMKs toward Cat-B, with the Arg derivative Bz-Phe-Arg-CH_2_F showing the highest second-order rate constant value (*k*_2nd_ = 390,000 M^−1^ s^−1^; Figure 2). The low reactivity of the m-PFMKs toward serine proteases was also confirmed.

Later on, Ahmed and co-workers synthesized a series of m-PFMKs in order to further investigate structure–activity relationship (SAR) of this class of compounds [16]. The dipeptide sequence Phe-Ala was kept constant, whereas the *N*-terminal group was extensively varied. They obtained potent m-PFMKs Cat-B inhibitors with *k*_2nd_ values that differ from each other by over 20-fold, suggesting that the *N*-terminal blocking group remarkably contributes to the interaction with the target. The most potent Cat-B inhibitor of this series turned out to be PhCH_2_OCOCH_2_CH_2_CO-Phe-Ala-CH_2_F with a second-order rate constant value of 21,000 M^−1^ s^−1^ (Figure 2). Besides for the potent Cat-B inhibition in vitro, this series of m-PFMKs were effective in reducing inflammation and degradation of articular cartilage and bone in an ex vivo model of adjuvant-induced arthritis, as it is well-known that rheumatoid arthritis is associated with high levels of Cat-B in the synovial joints [17].

The pharmacological profile of the lead m-PFMK, i.e., Z-Phe-Ala-FMK, was deeply investigated by Lawrence C.P. and co-workers, in particular its potential immunosuppressive properties on primary T cells [18]. The authors demonstrated that Z-Phe-Ala-FMK acts as an immunosuppressive agent via inhibition of the activation of T cells and by repressing their proliferation induced by mitogens and IL-2 in vitro. Furthermore, Z-Phe-Ala-FMK inhibits the processing of caspase-8 and caspase-3 to their respective subunits in resting T cells stimulated through the Ag receptor, but has no effect on the activation of these caspases during Fas-induced apoptosis in proliferating T cells. In vivo studies performed on a mouse model of pneumococcal disease (in which T cells play a leading role), showed also a significant increase of pneumococcal loads in both lungs and blood, confirming that Z-Phe-Ala-FMK is immunosuppressive both in vivo and in vitro.

Two other dipeptidic m-FMKs were synthesized and investigated more recently as cysteine proteases inhibitors. Specifically, Powers’ research group carried out a comparative study of m-PFMKs with other dipeptide-based compounds having different electrophilic warheads, such as vinyl sulfones, acyloxymethyl ketones, and diazomethyl ketones, as inhibitors of Cat-C [19]. This cysteine protease, also known as dipeptidyl peptidase I (DPP-I), is a lysosomal oligomeric exoprotease that processes and activates several serine protease zymogens within bone marrow derived cells (e.g., pro-granzymes of cytotoxic T cells and NK cells, pro-chymase of mast cells, and pro-cathepsin G of neutrophils) by removing dipeptides from their *N*-termini [20]. The two m-PKMKs, i.e., Gly-Phe-CH_2_F and Ala-Phe-CH_2_F, were potent inhibitors of Cat-C (*K*_i_ = 8.3 μM and *K*_i_ = 2.1 μM, respectively), but the inhibited enzyme regained most of its activity, indicating a reversible covalent inhibition mechanism.

The latest outcomes on m-PFMKs as Cats inhibitors come from the thorough study of Rudzińska M. et al. [21]. The authors developed two tetrapeptidyl m-FMKs, i.e., Ac-Pro-Leu-Val-Glu(OMe)-CH_2_F (Ac-PLVE-FMK) and Ac-Val-Leu-Pro-Glu(OMe)-CH_2_F (Ac-VLPE-FMK), on the basis of the well-known substrate Ac-PLVQ of the triticain-α, a wheat-derived (*Triticum aestivum*) cysteine protease of the papain-like family [22]. Both m-PFMKs were able to efficiently inhibit the activity of Cat-B and Cat-L in the target-based assay (human recombinant enzymes) and, more importantly, in the cell-based assay on two human renal cancer cell lines (769-P and A498), implying their ability to penetrate the cell membrane. Moreover, docking studies indicated (according to the results) that Ac-VLPE-FMK is a weaker inhibitor comparing to Ac-PLVE-FMK, as the Pro residue at P_2_ hinders the flexibility of the peptide and the consequent accommodation of the P_2_ side chain into the S_2_ hydrophobic pocket of the binding site [21]. By using these two m-PFMKs, the authors essentially explored the role of the aberrant expression of Cats in renal cancer [23], especially in relation to its aggressiveness and spreading, in such a way as to validate these lysosomal proteases as pharmacological targets. The two m-PFMKs did not significantly affect renal cancer cell viability. Conversely, the overall biology of the renal cancer cells (e.g., cell migration rate, cellular adhesion, colony formation, markers expression, anchorage-independent growth, etc.) and Cats expression were considerably influenced, indicating that the inhibition of Cats has a relevant impact on cancer cell phenotype [21].

### 2.2. Caspases Inhibitors

Caspases (Casps) are a large family of cysteine-dependent aspartate-directed endoproteases of fundamental importance for the maintenance of cellular homeostasis. They are mainly involved in programmed cell death (including apoptosis, pyroptosis, and necroptosis) and inflammatory processes [24]. On the basis on this functionality, the Casps family have been subdivided into apoptosis Casps (i.e., Casp-3, -6, -7, -8, and -9 in mammals) and inflammation Casps (i.e., caspase-1, -4, -5, and -12 in humans and Casp-1, -11, and -12 in mice) [25]. Casps are originally produced as monomeric units (pro-Casps), which undergo dimerization after signaling events leading to activation or inactivation of substrates, which in turn generate a cascade of apoptotic events or the production of pro-inflammatory cytokines. Dysregulation of Casps activity is related to several human diseases such as different types of cancer, degenerative diseases (e.g., Alzheimer’s disease), inflammatory disorders, and cardiovascular diseases. Therefore, the identification of specific Casps inhibitors constitutes a major focus of the current drug discovery efforts [26]. Since these endoproteases hydrolyze peptide bonds in correspondence to a P_1_ aspartic acid residue (which is recognized and anchored to the protein with the formation of a saline bridge), the vast majority of Casps inhibitor m-PFMKs have been designed with such a prerequisite in the peptide framework as a steady point. In some cases, the side chain free –COOH group of Asp at the P_1_ site was developed as a methyl ester group to enhance stability and cell membrane permeability of the related peptide. These m-PFMKs developed so far can be classified by the number of the amino acid residues that composes the peptide skeleton in monopeptides, dipeptides, tripeptides, and tetrapeptides.

In regard to monopeptidic m-FMKs, extensive research work has been performed on Boc-Asp(OMe)-FMK, a pan-Casp inhibitor which showed neuroprotection after systemic and local administration in a rat model of neonatal hypoxia–ischemia [27], and to prevent neuronal death in vitro through nerve growth factor rescue [28,29]. Moreover, Boc-Asp(OMe)-FMK was able to enhance the survival of spinal motoneurons in neonatal (but not in adult) rats after root avulsion of the C7 spinal cord [30]. Brown T.L. et al. proved that this m-PFMK completely blocks TGF-β-induced apoptosis (maintaining cellular viability) and αII-spectrin cleavage in WEHI 231 cells, and that these two events occur by at least two different apoptotic pathways that Boc-Asp(OMe)-FMK is able to discriminate [31,32]. Later on, Cowburn A.S. and co-workers demonstrated that TNFα-induced apoptosis in neutrophils is closely related to caspase activity, and that administration of Boc-Asp(OMe)-FMK can inhibit only TNFα-stimulated apoptosis with IC_50_ value of 39 μM [33]. In vivo tests performed by Clark R.S.B. et al. on rat models of traumatic brain injury highlighted that the local administration of Boc-Asp(OMe)-FMK delays (but does not prevent) neuron death via a mechanism involving Casps [34]. More in detail, the authors demonstrated that Boc-Asp(OMe)-FMK reduces the activity of the executioner Casp-3, and inhibits the initiator Casp-2 proteolysis and the release of mitochondrial cytochrome *c*, an effect that seems to be associated with the apoptotic activity of initiator Casps.

**Figure 3:**
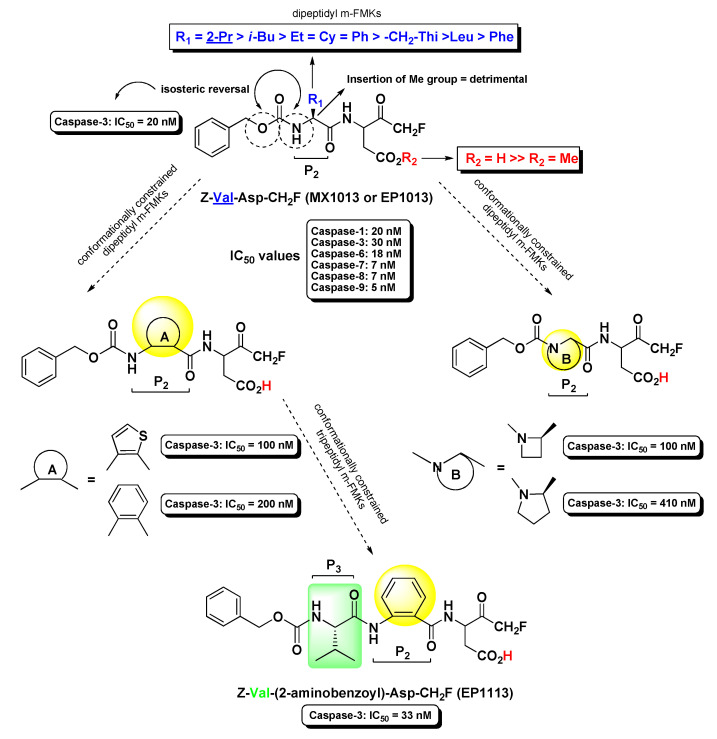
Chemical structures, progressive structure–activity relationship (SAR) analysis, and some biological results of the most relevant m-PFMKs developed by Wang Y. et al.

**Figure 4:**
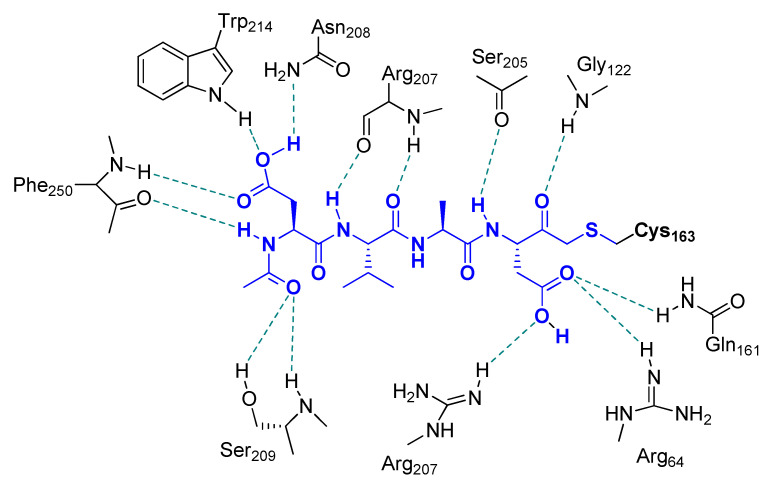
Main interactions observed for Ac-DVAD-FMK with Casp-3. The inhibitor was found covalently bound to the enzyme via formation of a thioether linkage with the side chain of Cys163.

Extensive work on dipeptidyl aspartyl m-FMKs with Casps inhibitory properties has been carried out by Wang Y. and co-workers with the aim of exploring the role of P_2_ amino acid on selectivity over Casps and other proteases. In a first SAR study, they identified Val as the best P_2_ amino acid for achieving activity toward Casp-3. Indeed, Z-Val-Asp-CH_2_F (also named MX1013 or EP1013) turned out to be a potent Casp-3 inhibitor with an IC_50_ value of 30 nM (Figure 3). Other P_2_ amino acids with hydrophobic side chains showed considerable potency toward this target, while the Z terminal group was kept constant. However, the same compound showed high selectivity only towards other cysteine and serine proteases, whereas the potency towards a panel of other Casps proved to be comparable or even higher (IC_50_ in the range 5–20 nM; Figure 3) than that recorded for Casp-3. Notably, the dipeptidic m-FMKs, having a P_1_ Asp residue with a free -COOH group, displayed inhibitory potency towards Casp-3 two order of magnitude higher than the methyl ester parent compounds. In addition, the introduction of an additional methyl at the P_2_ site turned out to be highly detrimental [35]. The same research group demonstrated that Z-Val-Asp-CH_2_F was able to exert potent in vivo cytoprotective activity in three rodent models of apoptosis by inhibiting three key markers of apoptosis, i.e., the proteolytic maturation of Casp-3, the Casp-3-mediated cleavage of PARP, and the fragmentation of genomic DNA [36]. As an extension of their SAR studies on this type of m-PFMKs, the authors replaced the P_2_ amino acid of the peptide framework with a series of *O*-protected (carbamoyl junction) peptidomimetic α-hydroxy acids. This replacement was planned on the basis of crystallographic studies carried out on tetrapeptidic inhibitors, which showed that P_2_ backbone nitrogen is not important for the interaction with the enzyme (see Figure 4) [37]. This modification resulted essentially is an isosteric NH/O reversal at the *N*-terminal group of the m-PFMKs, as pointed out in Figure 3. SAR outcomes of these new derivatives were similar to those observed in the previous series. They obtained potent (IC_50_ in the range 5–70 nM) pan-Casp inhibitors with high selectivity towards other non-Casp proteases, including the cysteine proteases Calpain I and Cat-B, and the serine protease coagulation factor Xa. Additional insights came from the aromatic end-group (which was extensively varied), with the halo-substituted phenyl ring providing the most potent Casp-3 inhibitors and the best cell apoptosis protection in vitro realistically due to the highest hydrophobicity that reflects a superior cell permeability. The phenylcarbamoyl derivative (named MX1135 or EP1135) was elected for further in vivo assessments showing good dose-dependent activity in a mouse liver apoptosis model [38]. SAR studies on these dipeptidyl m-FMKs continued by replacing the P_2_ amino acid with several 2-aminoaryl acids and other non-natural amino acids according to two types of modification of the peptide backbone (highlighted in yellow as A and B; Figure 3), both leading to conformationally constrained derivatives, which in principle are designed to increase metabolic stability and target selectivity [39]. All new series of dipeptidyl m-FMKs showed good inhibitory activity towards Casp-3 with IC_50_ values in the sub-micromolar to low-micromolar range. The two most potent derivatives of each type of peptidomimetics are reported in Figure 3. These four m-PFMKs were selected for HeLa cell apoptosis protection assays, which measure the protecting effects of Casp inhibitors against apoptosis induced by TNF-α, showing activity in the micromolar range. To further extend their SAR investigations, the authors added a P_3_ Glu residue at the *N*-terminus of one of the most active dipeptidyl m-FMKS (i.e., the type-A phenyl constrained derivative) in accordance with previous studies showing this substrate preference for Casp-3 inhibition [40]. However, this modification did not turn out to be fruitful as expected, indicating that the Glu side chain does not fit into the S_3_ pocket of the intended target. Surprisingly, the insertion of a Val residue at the P_3_ site led to a tripeptidyl m-FMK (i.e., Z-Val-(2-aminobenzoyl)-Asp-CH_2_F or EP1113; Figure 3) 6-fold more potent (IC_50_ = 33 nM) than the reference dipeptidyl m-FMK toward Casp-3 (though non-selective towards other Casps) and one order of magnitude more potent than the apoptosis protection agent [39].

**Figure 5:**
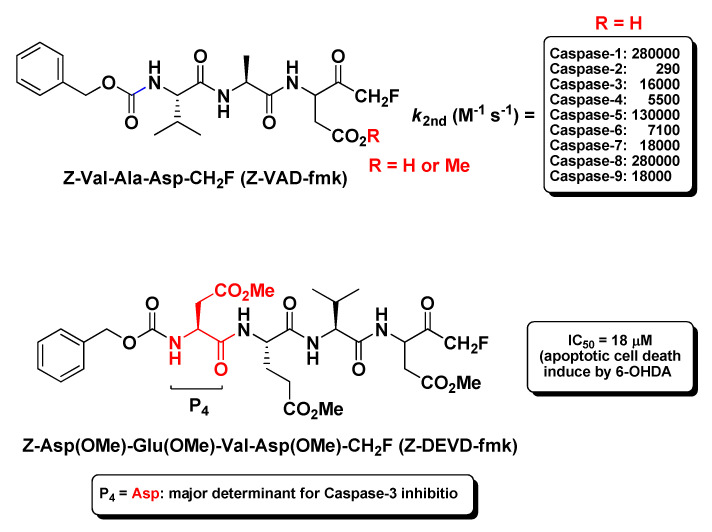
Chemical structure and some relevant biological data of the most representative tripeptidyl m-FMK (i.e., Z-VAD-fmk) and tetrapeptidyl m-FMK (i.e., Z-DEVD-fmk).

The prototype of the tripeptidyl m-FMKs endowed with specific Casp inhibitory properties is undoubtedly Z-Val-Ala-Asp-CH_2_F (better known as Z-VAD-fmk; Figure 5). This irreversible pan-Casp inhibitor showed second-order rate constants ranging from 290 M^−1^ s^−1^ for caspase-2 to 280,000 M^−1^ s^−1^ for Casp-1 and Casp-8 [32], and has been the object of several pharmacological studies on apoptosis. The first report of antiaptotic activity in vivo was provided by Rodriguez et al. in a mouse model of Fas-induced liver damage wherein Z-Val-Ala-Asp-CH_2_F prevented massive hepatocytes death after repeated intravenous injections within 1 h, and this protective effect is related to Casps inhibition [41]. Chandler J.M. and co-workers employed this m-PFMK as a tool to correlate the above-mentioned Fas-induced liver damage to the activation of the executioner Casp-3 and Casp-7, and to detect their subcellular localization [42]. Subsequently, other in vivo studies demonstrated that the methyl ester Z-Val-Ala-Asp(OMe)-CH_2_F is able to protect neurons from ischemic injury and from the excitotoxicity induced by the activation of glutamate receptor agonist AMPA and (to a lesser extent) NMDA [43,44,45,46], as well as to reduce myocardial ischemia–reperfusion injury attenuating cardiomyocyte apoptosis [47,48]. This m-PFMK showed also remarkable efficacy in another myocardial ischemia–reperfusion injury rat model wherein it limited the infarct size (24.6 ± 3.4%) when administered intravenously at 0.1 μM [49], in an intestinal ischemia–reperfusion mouse model, wherein it decreased the tissue injury when administered subcutaneously [50], and in a muscle ischemia–reperfusion mouse model [51]. In a model of renal ischemia, Z-Val-Ala-Asp(OMe)-CH_2_F prevented the early onset of renal apoptosis together with inflammation and tissue injury [52]. In previous in vivo studies, it showed efficacy in models of acute inflammation such as LPS-pyrexia and carrageenan edema, and this anti-inflammatory property had been ascribed to Casp-1 inhibition [53]. The selectivity of this m-PFMK for Casps was further ascertained by the Turk B. research group. However, in spite of its poor in vitro inhibitory activity profile towards a wide panel of papain-like proteases and legumain, Z-Val-Ala-Asp(OMe)-CH_2_F efficiently inhibited Cats activity in two cell-based apoptosis assays (i.e., Jurkat-T and HEK-293) at 100 μM, and specifically Cat-X and Cat-B activity in tissue extracts at concentrations as low as 1 μM, with IC_50_ values of 1.9 μM and 3.1 μM, respectively [54]. In a rabbit model of pneumococcal meningitis, Z-Val-Ala-Asp-CH_2_F inhibited neuronal cell death in the dentate gyrus of the hippocampus and reduced the inflammatory infiltrate into the cerebrospinal fluid. In this model it was demonstrated that Z-Val-Ala-Asp-CH_2_F produces protective effects by inhibiting apoptotic pathways initiated by host inflammatory factors [55]. Moreover, Z-Val-Ala-Asp-CH_2_F inhibited actinomycin D-induced apoptosis in Jurkat-T cells [56,57], exerted synergistic protective effects with bFGF towards brain injury in a transient focal ischemia model [58], and showed promise as potential therapeutics in apoptotic models of asthma [59] and multiple sclerosis [60]. Besides apoptosis models, this tripeptidyl m-FMK has been employed as a tool for several immunological studies in vivo, wherein it plays a role in controlling inflammation, cytokine production, and host innate immune response via Casps inhibition [61]. Shaalan A. et al. asserted this regulatory role to the ability of Z-Val-Ala-Asp-CH_2_F to interfere with TLR3 and IF gene expression in a mouse exocrine secretory tissue (submandibular glands) model after Casp-3 and Casp-8 inhibition [62]. Li X. and co-workers showed that Z-Val-Ala-Asp-CH_2_F can alleviate lipopolysaccharide-induced endotoxin shock in a mouse model by inducing necroptosis of macrophages and promote the aggregation of myeloid-derived suppressor cells [63]; Liu M. et al. evidenced that this broad-spectrum caspase inhibitor may reduce lung injury by significantly inhibiting the activation of myeloperoxidase, TNF-α, and IL-β in a rat model of severe acute pancreatitis [64]. Recently, a potential use for Z-Val-Ala-Asp-CH_2_F has also been suggested as a cryopreservative in the cattle industry for IVEP technology, since it has shown to prevent aberrant apoptosis and enhance cryotolerance of IVP bovine embryos via Casp-3 inhibition [65].

By increasing the length of the peptide sequence to four amino acid residues, more substrate specificity can be achieved towards Casps [66]. Since these tetrapeptidic compounds are endowed with lesser cell membrane permeability and bioavailability with respect to the tripeptidic analogues, all of them having amino acid residues with acidic side chain have been employed as methyl ester derivatives. The most exploited tetrapeptidyl m-FMK for pharmacological or biochemical assessments has been Z-Asp(OMe)-Glu(OMe)-Val-Asp(OMe)-CH_2_F (also reported as Z-DEVD-fmk; Figure 5). The P_4_ Asp residue is the major determinant for its relative selectivity to Casp-3. As a matter of fact, a similar tetrapeptidyl m-FMK, i.e., Ac-Asp(OMe)-Val-Ala-Asp(OMe)-CH_2_F (Ac-DVAD-fmk), was used to determine the 3D-structure of this Casp isoform by X-ray crystallography and its topological similarity with interleukin-1β-converting enzyme (ICE, Casp-1) [37]. As for the broad-spectrum Casp inhibitor Z-VAD-fmk, Z-DEVD-fmk turned out to be effective in reducing the infarct size, behavioral deficit, and excitotoxicity in several murine models of focal cerebral ischemia after i.c.v. administration [43,44,46,58,67,68,69], underlining the key role of Casp-3 during the apoptotic processes. However, this neuroprotective role of Z-DEVD-fmk has been brought into question more recently in a rat model of global ischemia caused by cardiac arrest, wherein i.c.v. administration of Z-DEVD-fmk did not affect neurological outcome and neuronal cell death [70]. Besides, this tetrapeptidic m-FMK showed protection against traumatic brain injury both in vitro and in vivo [71], as well as against oxidative stress in cell cultures after starving, radiation, and calcium or potassium overload [72]. Kanthasamy A.G. et al. proved the protective effects of Z-DEVD-fmk in an experimental cell culture model of Parkinson’s disease, wherein it reduced cytotoxicity, Casp-3 activation, and DNA fragmentation in N27 cells after co-administration of Parkinsonian toxins MPP^+^ and 6-OHDA [73]. The neuroprotection against the dopaminergic degeneration exerted by this m-PFMK (IC_50_ = 18 μM; apoptosis induced by 6-OHDA only; Figure 5) was compared with that of a more substrate-specific tetrapeptidyl m-FMK, i.e., Z-Asp(OMe)-Ile-Pro-Asp(OMe)-CH_2_F (Z-DIPD-fmk; IC_50_ = 6 μM), developed by the authors, as Casp-3 cleaves between Pro and Asp residues and activates PKCδ, a member of the PKC isoform family that plays a key role in apoptosis of dopaminergic neurons [74].

Other tetrapeptidyl m-FMKs employed as target-specific tools in pharmacological studies were the Casp-8 inhibitor Z-Ile-Glu(OMe)-Thr-Asp(OMe)-CH_2_F (Z-IETD-fmk) and the Casp-9 inhibitor Z-Leu-Glu(OMe)-His-Asp(OMe)-CH_2_F (Z-LEHD-fmk), which were effective in the myocardial ischemia–reperfusion injury rat model developed by Mocanu M.M. et al. in which they limited the infarct size of 23.0 ± 5.4% and 19.3 ± 2.4 5, respectively, when administered intravenously both at 0.07 Μm [49]. In the selectivity study carried out by Turk B. et al. (see hereinabove for the tripeptidic derivative Z-VAD-fmk), the Casp-1, -4, and -5 inhibitor Z-Tyr-Val-Ala-Asp(OMe)-CH_2_F (Z-YVAD-fmk) and the standard Casp-3 inhibitor Z-DEVD-fmk showed inhibitory activity also against Cats in cell-based assays (i.e., Jurkat-T and HEK-293) at high concentrations (100 μM), as well as specific Cat-X and Cat-B inhibitory activity in tissue extracts at concentrations as low as 1 μM, with IC_50_ values of 0.65 μM and 0.63 μM for Cat-X, and of 1.4 μM and 0.62 μM for Cat-B, respectively [54].

### 2.3. Calpain(s) Inhibitors

**Figure 6:**
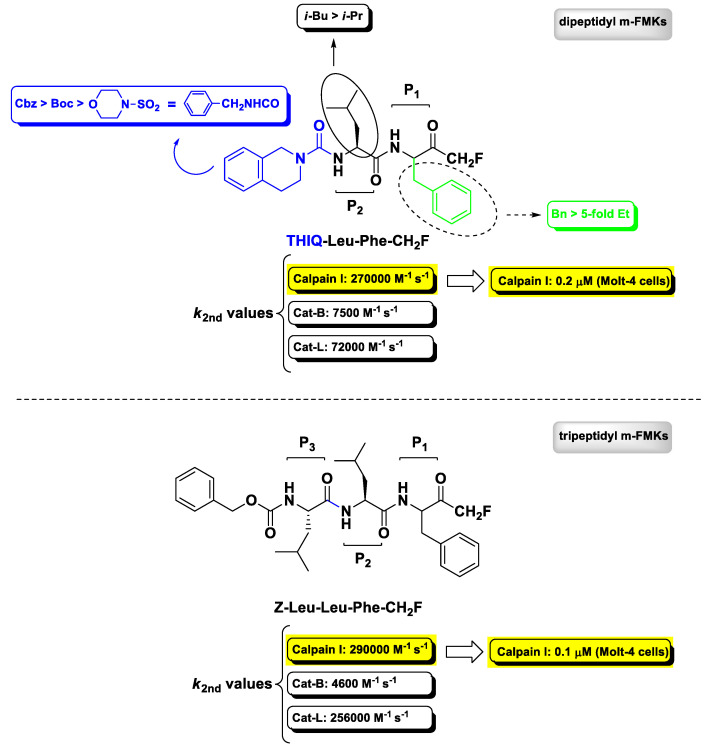
Chemical structure, SAR analysis, and biological activity of the most relevant m-PFMKs developed by Chatterjee S. et al. mainly as calpain I inhibitors.

Calcium-activated neutral proteases (calpains) are non-lysosomal intracellular cysteine proteases belonging to the papain superfamily of proteolytic enzymes expressed ubiquitously in mammals and other organisms wherein they play a role in many regulatory processes both in physiological and pathological conditions, such as apoptosis, cell motility, and cellular proliferation. Calpain I (also known as μ-calpain) is the most studied isoform, as it is the predominant form activated at near-micromolar Ca^2+^ levels during pathological conditions related to the central nervous system and therefore identified as one of the most promising druggable target for the treatment of neurodegenerative diseases. Calpain II (m-calpain) is the second most known isoform, which requires higher Ca^2+^ levels (millimolar) to be activated [75]. The first report on m-PFMKs as calpain inhibitors was provided in 1992 by Shaw E. et al. wherein the authors showed that the dipeptide Z-Leu-Tyr-CH_2_F and the tripeptide Z-Leu-Leu-Tyr-CH_2_F were effective in irreversibly inactivating in vitro chicken gizzard calpain II with second-order rate constant values of 17,000 M^−1^ s^−1^ and 28,900 M^−1^ s^−1^, respectively. However, these m-PFMKs did not show much selectivity, as they were comparably or ever more potent towards Cat-L [76]. A more exhaustive SAR analysis about the dipeptidyl derivatives having a m-FMK warhead was provided, later on, by Chatterjee’s research group in 1996/97 [77,78]. While previous studies had shown that calpain(s) cleave at peptide bonds of amino acids preceded by a Leu residue and therefore Leu or other branched amino acids (e.g., Val) are preferred at the P2 site [79], Chatterjee’s works showed that the P_1_ side chain and the *N*-terminal capping group have a notable effect on the potency and selectivity towards recombinant human calpain I. This SAR analysis is summarily depicted in Figure 6, wherein is also reported the obtained most potent (calpain I: *k*_2nd_ = 270,000 M^−1^ s^−1^) and selective (Cat-B: *k*_2nd_ = 7500 M^−1^ s^−1^; Cat-L: *k*_2nd_ = 72,000 M^−1^ s^−1^) dipeptidyl m-FMK, bearing a Phe residue at the P_1_ site and a tetrahydroisoquinolyl moiety at the *N*-terminus (i.e., THIQ-Leu-Phe-CH_2_F), which is still considered the lead calpain I inhibitor of this class of derivatives [78]. The tripeptidyl derivative Z-Leu-Leu-Phe-CH_2_F (Figure 6), synthesized by the same authors, turned out to be equipotent to THIQ-Leu-Phe-CH_2_F as calpain I inhibitor (*k*_2nd_ = 290,000 M^−1^ s^−1^) in the enzymatic assay (although less selective towards Cat-L), while both compounds potently inhibited intracellular calpain I in an intact cell assay (Molt-4 cells) with IC_50_ values of 0.1 μ and 0.2 μM, respectively [77].

### 2.4. N-Glycanase Inhibitors

Peptide:*N*-glycanase (PNGase, NGLY1, or simply *N*-glycanase) is a cytosolic cysteine protease involved in numerous proteolytic processes. Its main activity, closely related to that of the proteasome, is to degrade misfolded/unassembled proteins by acting as a deglycosylating enzyme of *N*-linked glycoproteins, and it is part of a multistep process aimed at regulating the quality of the newly synthesized glycoproteins in the endoplasmic reticulum (ER) called ER-associated degradation. The unique proteolytic activity of this cysteine protease is entrusted to a catalytic triad Cys–His–Asp, which cuts at the level of β-aspartyl glucosamine bonds and releases free glycans and deglycosylated peptides in which the *N*-glycosylated Asn residues are converted into Asp residues [80]. In regard to m-PFMKs, *N*-glycanase is effectively inhibited by the tripeptidyl derivative pan-Casp inhibitor Z-Val-Ala-Asp(OMe)-CH_2_F (Z-VAD-fmk; Figure 5) [81,82] through the formation of a covalent bond between the carbonyl of the m-FMK moiety and the –SH group of the Cys191 residue [83,84]. Misaghi S. et al. demonstrated that the inhibition of yeast and mammalian *N*-glycanase occurs at concentrations lower than those commonly used for Casp inhibition in vivo and employed Z-VAD-fmk as a probe to clarify the role of this enzyme in glycoprotein turnover [82]. A fluorescent derivative of this m-PFMK instead, i.e., BODIPY-VAD-fmk, was employed by Witte M.D. and co-workers for the rapid identification of new chitobiose-based *N*-glycanase inhibitor by means of competition assays [84].

### 2.5. Sentrin/SUMO-Specific Proteases Inhibitors

Post-translational modification of proteins by ubiquitin and SUMOs (small ubiquitin-like modifiers) is a versatile and reversible process devoted to the homeostasis of intracellular proteins [85]. As for the ubiquitin pathway, where the deubiquitination occurs by means of specific enzymes (i.e., DUBs), SUMOs are removed by their substrates by the action of sentrin/SUMO-specific proteases named SENPs. Besides this isopeptidase activity, SENPs are also endowed with endopeptidase activity directed to the removal of the *C*-terminal extension from immature SUMOs [86].

**Figure 7:**
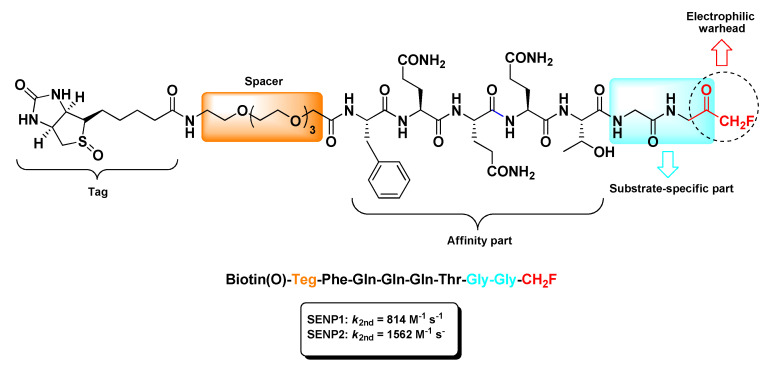
Chemical structure and biological activity of the biotinylated activity-based probe bearing a m-FMK *C*-terminal warhead developed by Funeriu’s research group.

The sole m-PFMKs targeting the catalytic site (Cys residue) of SENPs was developed by Funeriu’s research group as a biotinylated selective activity-based probe [87]. According to crystallographic studies [88,89], this m-PFMK was designed with a -Gly-Gly-CH_2_F dipeptidic *C*-terminal warhead that accommodates to the narrow tunnel-like cavity of the SENP active site and a remaining peptide sequence suitable with the SUMO–SENP complexes. The resulting probe, i.e., biotin(O)-Teg-Phe-Gln-Gln-Gln-Thr-Gly-Gly-CH_2_F (Figure 7), was conjugated to biotin through a tetra(ethylene glycol) (Teg)-spacer and irreversibly tied to SENP1 (*k*_2nd_ = 814 M^−1^ s^−1^) and SENP2 (*k*_2nd_ = 1562 M^−1^ s^−1^) at low-micromolar to high-nanomolar concentrations. Moreover, in vitro labeling studies confirmed that this m-PFMK out-competes with SUMO1 for the formation of the reversible complex SUMO1–SENP1, indicating that the fluorinated probe and SUMO1 share a common binding site on SENP1 [87].

### 2.6. Protozoan Cysteine Proteases Inhibitors

**Figure 8:**
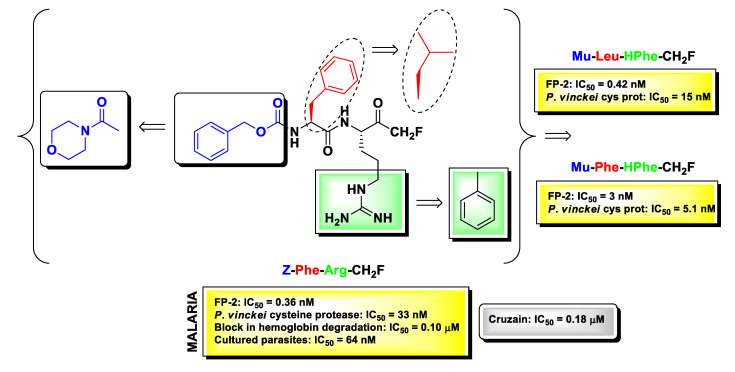
Chemical structure of the most relevant m-PFMK as protozoan cysteine protease inhibitor (Z-Phe-Arg-CH_2_F), together with biological activity and chemical modifications in relation to its antimalarial activity.

m-PFMKs were also employed in the early 1990s as compelling pharmacological tools to clarify the role of cysteine proteases in the lifecycle on protozoan parasites, which are responsible still today for deadly infectious diseases in tropical and subtropical regions of Africa, South America, and Asia. Extensive studies in this context have been carried out by Rosenthal’s research group. The authors examined the antimalarial effects of a panel of dipeptidyl m-FMKs by testing at the same time their ability to inhibit *Plasmodium falciparum* falcipain-2 (FP-2; a papain-like cysteine protease formerly named *P. falciparum* trophozoite cysteine proteinase-TCP), to block hemoglobin degradation and to kill cultured parasites [90]. FP-2 is nowadays a recognized enzymatic target for the drug design and discovery of new antimalarial compounds as it plays key roles at different stages of the parasite development such as hemoglobin digestion (early trophozoite stage) and rupture of cytoskeletal elements of the erythrocyte membrane (schizont stage) [91]. From the Rosenthal’s studies emerged the understanding that Z-Phe-Arg-CH_2_F (Figure 8) was by far the most potent derivative, showing IC_50_ values in the sub-nanomolar (IC_50_ = 0.36 nM) range in the enzymatic assay, sub-micromolar (IC_50_ = 0.10 μM) in the hemoglobin degradation assay, and nanomolar (IC_50_ = 64 nM) in the parasite viability assay performed on four different strains of *P. falciparum* (Itg2, FCT3, W-2, and D6). Furthermore, Z-Phe-Arg-CH_2_F was non-toxic at micromolar concentrations towards four different human cell lines, i.e., foreskin fibroblasts, MRC-5, Int 407, and Flow 2000 [90,92]. SAR studies on this lead m-PFMK were extended by Rosenthal and co-workers, and the most significant modifications to it are highlighted in Figure 8. By introducing the *N*-carbonyl morpholine group at the *N*-terminus of the dipeptide framework in place of the benzyloxycarbonyl (Z) group, they obtained various compounds still highly active against FP-2 but with a good inhibitory activity also against *P. vinckei* cysteine protease, an enzyme that shares several biochemical properties with FP-2 such as substrate specificity, pH optimum, molecular mass, etc. Further optimizations in terms of enzyme inhibition were obtained by switching the Phe residue at P_2_ with a Leu residue and by introducing a HPhe residue at P_1_ in place of the Arg residue. The derivative Mu-Phe-HPhe-CH_2_F was selected for further assessments in vivo in a murine malaria model wherein it elicited long-term cures in 80% of the treated animals [92].

The dipeptidyl m-FMKs Z-Phe-Arg-CH_2_F (Figure 8) and Z-Phe-Ala-CH_2_F (Figure 2) were also evaluated by Harth G. et al. as inhibitors of the activity of the major cysteine protease of *Trypanosoma cruzi*, that is cruzain (also referred to as cruzipain, the full-length native enzyme). This enzyme is a Cat-L-like protease expressed in all stages of the parasite lifecycle, and it is considered one the primary therapeutic targets for the treatment of Chagas’ disease (also known as American trypanosomiasis) [93]. In this study the authors showed that both m-PFMKs inhibit cruzain with IC_50_ values of 0.18 μM and 2.2 μM, respectively, and that this inhibition has dramatic effects on the replication of all parasite stages, in particular during the intracellular transition trypomastigote–amastigote–trypomastigote, with minimal toxicity to mammalian cells (J774, LLC-MK_2_, and ATCC) [94].

**Figure 9:**
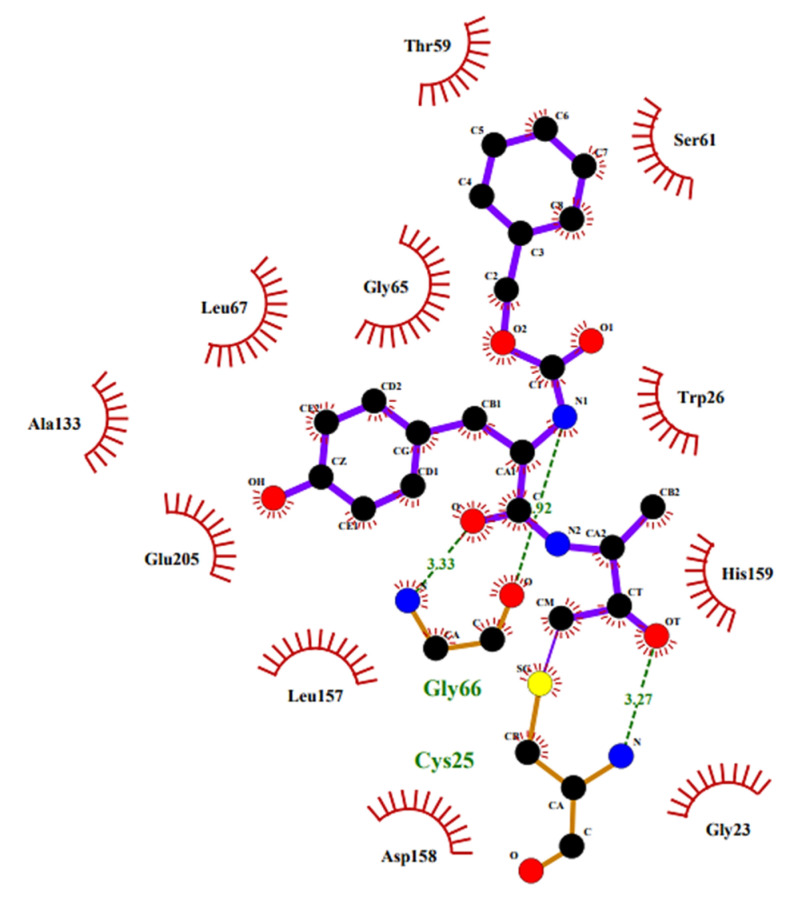
The 2D structure of Z-Tyr-Ala-CH_2_F in complex with cruzain. The 2D plot was generated by LigPlot+ (2.1, EMBL-EBI, Wellcome Genome Campus, Hinxton, Cambridgeshire, United Kingdom); hydrogen bonds are shown as green dotted lines, while the spoked arcs represent residues making nonbonded contacts with the ligand.

To elucidate the binding of these PFMKs, Gillmor S. et al. determined the crystal structure of Z-Tyr-Ala-CH_2_F in complex with cruzain, and the 2D structure showing the main interactions is depicted in Figure 9 [95].

### 2.7. SARS-CoV M^pro^ and Other Viral Proteases Inhibitors

Severe acute respiratory syndrome coronavirus (SARS-CoV) is an RNA virus spread worldwide from China in 2002 and belonging to the β-CoVs, one of the four genera of CoVs [96]. Like other members of the *Coronaviridae* family, SARS-CoV uses two cysteine proteases to process viral polyproteins and convert them into their mature form, a fundamental step for the CoV replication cycle [97]. Therefore, the inhibition of these proteases, namely SARS-CoV main protease (M^Pro^) and papain-like protease (PL^pro^), represents a valid strategy to tackle the infection. In this regard, SARS-CoV M^Pro^ (also called 3CL^pro^ being a chymotrypsin-like protease) is the most studied and topical targets [98], also in the view that the SARS-CoV-2 (the causative agent of the COVID-19) M^pro^ shows significant genetic similarities and behavior pattern to the SARS-CoV M^pro^ [99,100].

**Figure 10:**
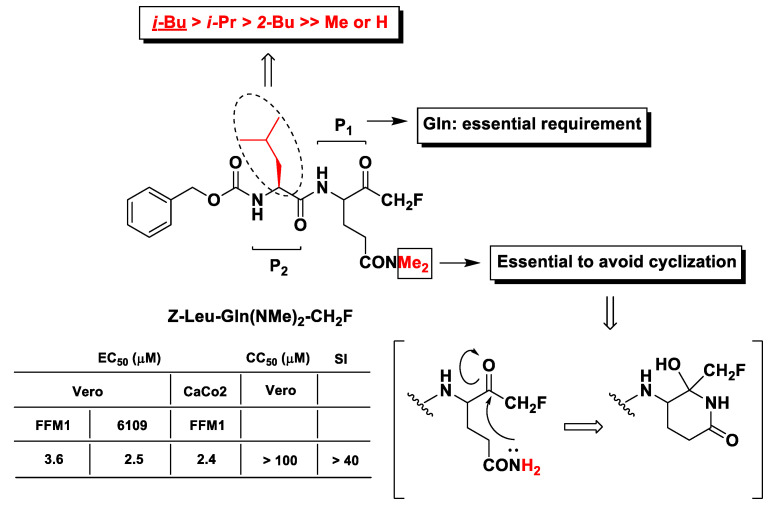
Chemical structures, SAR analysis, and biological results (protection from cytopathic effect in infected cells) of the most relevant m-PFMKs developed by Zhang H.-Z. et al.

The first evidence of m-PFMKs as potential SARS-CoV M^pro^ inhibitors was provided by Zhang H.-Z. et al. through the synthesis of a panel of dipeptidyl *N*,*N*-dimethyl glutaminyl FMKs and their antiviral activity assessment by cytopathic effect inhibition in SARS-CoV infected Vero and CaCo2 cells [101]. The most active derivative of this series of m-PFMKs turned out to be Z-Leu-Gln(NMe_2_)-CH_2_F, which showed efficacy in protecting cells with EC_50_ values in the low-micromolar range with no toxicity in healthy cells and a SI of > 40 (Figure 10). SAR analysis indicated that the Gln residue at P_1_ is essential for the recognition by the protease. Both *N*-hydrogens of the Gln side chain were replaced with two methyl groups in order to abolish the nucleophilic property of the Gln –NH_2_ group and avoid the formation of the biologically inactive six-terms lactam structure (Figure 10), as observed for analogous m-PFMKs [102]. Moreover, the presence of the Leu hydrophobic side chain at P_2_ seems to be also important for the activity. Indeed, its replacement with an H or Me group considerably reduces the cytoprotective effect of this compound, whereas the replacement with other alkyl side chains is tolerated, although it leads to a partial reduction in potency.

SARS-CoV M^pro^ is functionally analogous and exhibits similarity of cleavage site specificity to that of the main protease of picornaviruses 3C^pro^, a large family of viruses that also are responsible for respiratory illnesses [98]. However, the only report on m-PFMKs endowed with inhibitory activity towards proteases of the picornaviral family was provided by Morris T.S. et al. and pertains to the irreversible inhibition of hepatitis A virus (HAV) 3C^pro^ by the tetrapeptidyl derivative Ac-Leu-Ala-Ala-Gln(NMe)_2_-CH_2_F, with a second-order rate constant of 3300 M^−1^ s^−1^. This m-PKMK turned out to also be effective in ex vivo studies (FRhK-4-cells) in which it was able to reduce by 25-fold the progeny virus production when administered at 5 μM 24 h post-infection [102].

## 3. Peptidyl di-Fluoromethyl Ketones (d-PFMKs)

**Figure 11:**
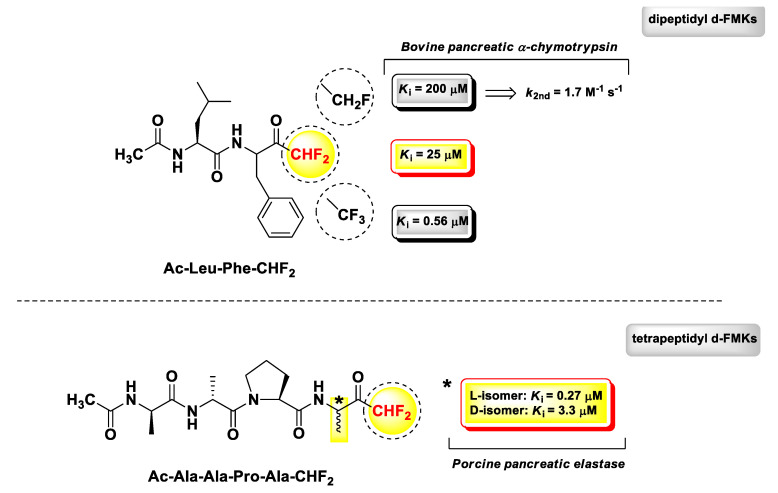
Chemical structures, biological activity, and comparative analysis of most relevant d-PFMKs developed by Imperiali B. and Abeles R.H.

d-PFMKs are a class of compounds still little explored due to the complexity of the chemistry concerning the construction of the d-FMK moiety [103,104], which becomes much more challenging when applied to peptidic substrates. One of the most extensive works in this regard has been performed by Imperiali B. and Abeles R.H. in a comparative study on PFMKs as inhibitors of serine proteases [105]. They synthesized a series of fluorinated derivatives and assessed their biological activity towards two serine proteases, i.e., bovine pancreatic α-chymotrypsin and porcine pancreatic elastase. Peptide aldehydes were used as reference compounds, since it was assumed that they form stable hemiacetal similarly to the FMKs by reacting with the γ-OH group of the catalytic serine residue. The trend of enzyme inhibitory properties of these PFMKs was explored by changing the length of the peptide sequence and the number of fluorine atoms at the FMK moiety. The two most representative d-PFMKs of this series of derivatives are reported in Figure 11, together with the SAR insights that emerged from this study. Among the dipeptidic derivatives, mainly designed as α-chymotrypsin inhibitors, the most active compound turned out to be Ac-Leu-Phe-CHF_2_ (*K*_i_ = 25 μM), in accordance with the known substrate preference of the enzyme, which requires aromatic amino acids at P_1_ and amino acids with hydrophobic side chains at the P_2_ for an effective cleavage [106]. By increasing the number of fluorine atoms at the FMK moiety, the binding affinity for the target improved considerably. However, mechanistic and kinetic parameters must be taken into account for a proper comparison. For instance, Ac-Leu-Phe-CHF_2_ acted as a reversible competitive inhibitor (consistent with high percentage of the hydrated form of the FMK moiety), whereas the trifluoromethyl analogue (*K*_i_ = 0.56 μM) exerted a slow-binding reversible competitive inhibition. The monofluoromethyl analogue, instead, was a poor inhibitor (*K*_i_ = 200 μM) of α-chymotrypsin and showed also an irreversible time-dependent inhibition (*k*_2nd_ = 1.7 M^−1^ s^−1^), as demonstrated by ^19^F NMR spectroscopy [105]. Therefore, di- and tri-fluoro derivatives were used as substrates for subsequent optimizations.

Since the recognition surface of the elastase-like proteases are far more extended with respect to those of trypsin- and chymotrypsin-like proteases, the authors determined to develop tetrapeptide derivatives for the assessments on porcine pancreatic elastase. As for the dipeptidic derivatives, the peptide sequence of the tetra-derivatives was chosen on the basis of previous substrate specificity studies [107]. As expected, the transition from dipeptide to tetrapeptide led to a high increase in the inhibitory activity. It is noteworthy that in this case, a significant difference was not detected in terms of activity between d-PFMKs and t-PFMKs. The most active derivative was Ac-Ala-Ala-Pro-Ala-CHF_2_ (Figure 11), for which it was also demonstrated the importance of the stereochemistry at the P_1_ site after HPLC separation of the two constituent diastereomers. The isomer with the P_1_ residue with l-configuration turned out to be ~12-fold more active than the isomer with d-configuration at the same position. Furthermore, the mechanism associated with the inhibition of elastase resulted in a dual reversible competitive mode, i.e., rapid- and slow-binding.

**Figure 12:**
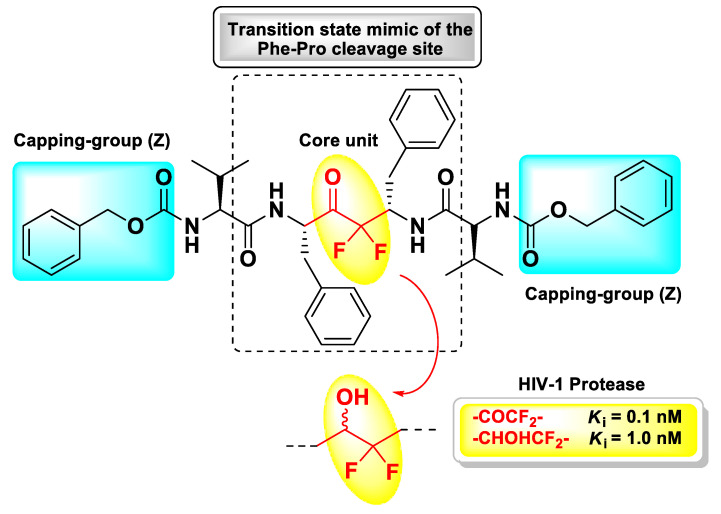
Chemical structure and biological activity of the symmetrical pseudo-peptide developed by Sham H.L. et al. as inhibitor of HIV-1 protease.

Since the d-FMK moiety can easily hydrate forming stable *gem*-diols and then mimicking the hydrolysis transition-state, another interesting application of this functional group has been its insertion as internal core unit of peptidomimetics designed as aspartic protease inhibitors. In particular, Sham H.L. et al. applied this strategy for the structure-based development of a *C*_2_ symmetric HIV-1 protease inhibitor [108]. This retroviral aspartic protease is essential for the HIV replication cycle and plays a fundamental role in the maturation process of viral polyproteins (namely, Gag, and Gag-Pol) by cleaving Phe–Pro bonds [109]. Therefore, it is considered one of the primary targets for the treatment of AIDS. Previous crystallographic studies have shown that HIV-1 protease functions as a *C*_2_ symmetric homodimer consisting of two identical 99 amino acid subunits [110,111], hence the concept of designing the novel inhibitor with a symmetric structure. The d-FMK unit was inserted as central core within a pseudo-symmetrical tetrapeptidic motif Val–Phe–Phe–Val, with both *N*-termini protected with the Z group and the functionalized inner dipeptide framework intended as a transition-state mimic of the Phe–Pro cleavage site of the HIV-1 protease (Figure 12). The resulting compound turned out be an extremely potent (*K*_i_ = 0.1 nM) inhibitor of the target, 10-fold higher than the hydroxyl parent compound (*K*_i_ = 1.0 nM) [108].

The principle of mimicking the transition-state hydrolysis by introducing d-FMK moieties within a peptide framework was also widely exploited in drug design to develop other aspartyl and serine protease inhibitors, in particular di-fluorostatine and di-fluorostatone containing pseudo-peptides [112]. However, a thorough discussion of this topic is beyond the scope of this review, which essentially deals with PFMKs as C-terminal electrophilic warheads.

## 4. Peptidyl tri-Fluoromethyl Ketones (t-PFMKs)

### 4.1. Elastase Inhibitors

**Figure 13:**
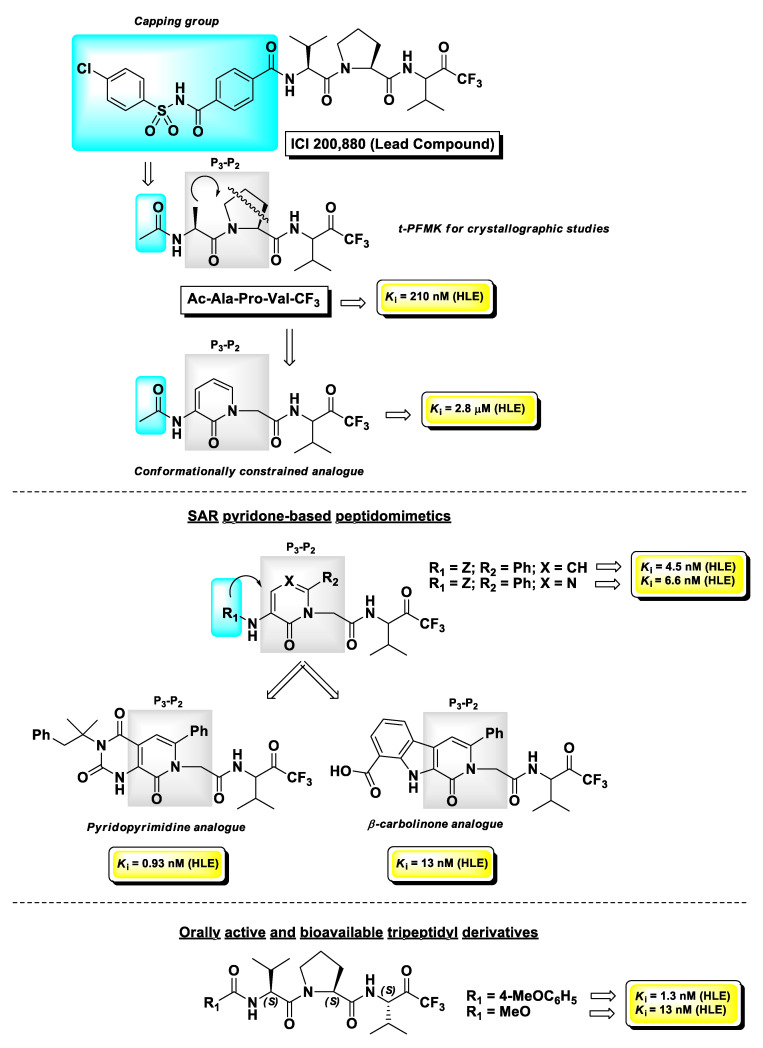
Chemical structures, progressive SAR analysis, and *K*_i_ values towards human leukocyte elastase (HLE) of the most relevant t-PFMKs developed by Brown F.J. and co-workers.

**Figure 14:**
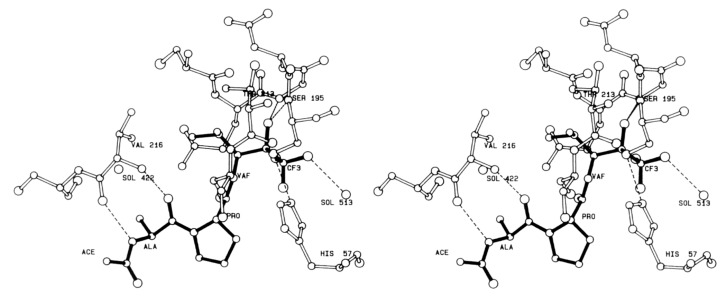
A stereo view of the inhibitor Ac-Ala-Pro-Val-CF_3_ in the extended binding site of porcine pancreatic elastase [116].

Human leukocyte elastase (HLE), also known as human neutrophil elastase (HNE), is a serine protease involved in chronic inflammatory processes. In particular, it is produced by polymorphonuclear leukocytes in response to inflammatory stimuli and its overexpression and high activity are believed to lead to various uncontrolled effects such as mucus hypersecretion and impaired host defense. This altered physiological pattern is associated with the pathogenesis of pulmonary emphysema, chronic bronchitis, and cystic fibrosis [113]. The development of HLE inhibitors could therefore be useful in the treatment of these chronic pathologies. Among the peptide-based inhibitors of this enzyme bearing a t-FMK warhead, the tripeptidyl derivative ICI 200,880 (Figure 13) has been the most representative, since it reached pre-clinical trials as a long-lasting protective agent against HLE-induced lung damage [114] and also showed protective effects associated with decreased neutrophil infiltration in animal models of myocardial ischemia–reperfusion dysfunction [115]. Starting from this lead structure, Brown F.J. and co-workers indirectly demonstrated by crystallographic studies carried out by Takahashi L.H. et al. the binding mode of this t-PFMK to HLE by using one of their ICI 200,880 analogues, i.e., Ac-Ala-Pro-Val-CF_3_ (Figure 14), in complex with the HLE-related enzyme porcine pancreatic elastase [116]. This X-ray diffraction analysis evidenced that the t-FMK group inserted deep inside the “oxyanionic” cavity of the target, presumably in the form of alkoxide, was stabilized by hydrogen bond interactions with the nitrogen atoms of Gly193 and Ser195. Moreover, it has been observed that the Ser195 -OH group of the enzyme forms a stable tetrahedral adduct with the inhibitor, and that α-lower alkyl amino acids (e.g., Val, Leu) are preferred at the P_1_ site. At the P_2_ site instead, the presence of a Pro residue (although it leads to an increase in conformational restriction) did not contribute any hydrogen bonds to the antiparallel β-pleated sheet arrangement (Figure 14).

Taking into consideration that both tripeptidyl derivatives were poorly active for oral administration, they proposed to reduce the peptidic character of the backbone of these inhibitors by introducing a pyridin-2(*H*)-one scaffold as a conformationally constrained achiral mimetic of the P_3_-P_2_ portion of the t-PFMKs. The design of these peptidomimetics led to HLE inhibitors with significant binding affinity for the target with *K*_i_ values in the low-micromolar to sub-micromolar range. Further optimizations of this series of derivatives were obtained by introducing a phenyl group at C6 of the pyridone nucleus and by varying length and chemical features of the *N*-terminal capping group, providing t-PFMKs with *K*_i_ values in the low-nanomolar range. Concurrently, the same research group developed peptidomimetics containing other pyridone-based scaffolds such as pyrimidone, pyridopyrimidine, and β-carbolinone analogues, obtaining in some cases compounds with *K*_i_ values in the sub-nanomolar range [117]. The most explored series was the pyrimidone-based one, in a comparative study with peptidomimetic analogues having boronic acid as the electrophilic warhead. Although overall the boronic acid derivatives were more potent inhibitors of HLE in vitro, the corresponding t-FMKs showed a superior pharmacological profile after oral administration, in an in vivo hamster model of acute hemorrhage induced by a subsequent intratracheal challenge of HLE. Moreover, X-ray crystallographic studies confirmed that the pyrimidone scaffolds act as a β-turn mimetic [118]. The structure of one of the best candidates of this series of derivatives is depicted in Figure 13. However, later on the same research team developed tripeptidyl derivatives bearing a t-FMK warhead with a superior pharmacological profile with respect to the pyrimidone-type FMKs, by carrying out a synthetic methodology that allowed them to obtain compounds in optically pure form [119]. The two hit compounds of this series with excellent oral activity and bioavailability are also depicted in Figure 13.

**Figure 15:**
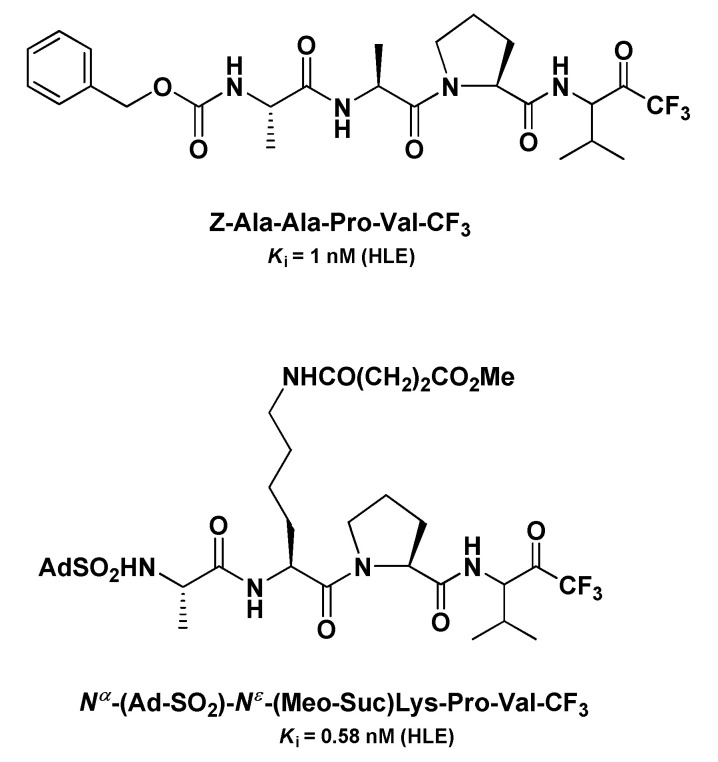
Chemical structures and *K*_i_ values towards HLE of the two most relevant t-PFMKs developed by Peet N.P. et al.

Another panel of very potent t-PFMKs as HLE inhibitors was developed by Peet N.P. et al. in the early 1990s [120]. Among the tetrapeptidyl derivatives with an Ala–Ala–Pro–Val framework, it turned out that the Z *N*-protecting group confers the highest potency (*K*_i_ = 1 nM), whereas within the tripeptidyl series, the derivative *N^α^*-(Ad-SO_2_)-*N^ε^*-(MeO-Suc)Lys-Pro-Val-CF_3_ was by far the most active (*K*_i_ = 0.58 nM) (Figure 15). For these inhibitors, the authors demonstrated also that their potency is higher when added to the enzyme solution from a stock solution of DMSO than when they are added in a buffer equilibrated prior to the assay, suggesting that the carbonyl form of the FMK moiety is likely the more active.

### 4.2. Chymase Inhibitors

**Figure 16:**
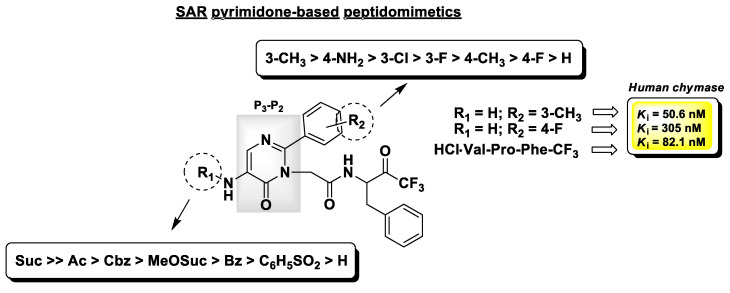
Chemical structure, partial SAR analysis, and *K*_i_ values towards human chymase of the t-PFMKs developed by Akahoshi F. and co-workers.

Chymases are serine proteases mainly produced by mast cells and play an important role in the response to inflammatory stimuli. The involvement of chymases in inflammatory processes, such as asthma and allergy, is due to their ability to provoke the infiltration of inflammatory cells and their consequent release of histamine and other mediators. They are also responsible for the production of Angiotensin II in the cardiovascular system, and an overexpression of these enzymes has been found in the human atherosclerotic aorta and detected in other cardiovascular diseases [121]. As for elastases, this family of serine proteases may be selectively inhibited by t-PFMKs. Crystallographic studies have demonstrated that, at variance with HLE, human chymase prefers aromatic amino acids such as Phe or Tyr at the P_1_ site [122]. On this basis, and in analogy to that proposed by Veale C.A. et al. for HLE inhibitors (see herein-above) [118], Akahoshi F. and co-workers used the tripeptidic sequence Val–Pro–Phe as reference motif and synthesized a set of orally active pseudo-peptidyl t-FMKs with a pyrimidinone scaffold as isosteric replacement of the Val–Pro dipeptidic fragment at the P_3_-P_2_ site [123]. The most relevant SAR insights of this series of chymase inhibitors are reported in Figure 16, together with the *K*_i_ values of the most potent and selective derivative (*K*_i_ = 50.6 nM), the reference peptidic analogue (*K*_i_ = 82.1 nM), and the compound which showed the best pharmacokinetic parameters after oral administration in rats (*K*_i_ = 305 nM).

### 4.3. Histone Deacetylase Inhibitors

The high propensity of the t-FMK moiety to form *gem*-diols in aqueous media has been exploited in drug discovery for the design of molecules able to inhibit metalloproteases by chelating their metal cofactor [124]. Histone deacetylases (HDACs) are a class of Zn^2+^-dependent proteases which essentially remove acetyl groups from *N^ε^*-acetyl Lys residues on a histone, allowing these proteins to wrap the DNA more tightly. HDACs are well recognized epigenetic regulators, and their uncontrolled activity has been associated with the genesis and progression of several types of malignancy [125]. Therefore, the development of HDAC inhibitors constitutes a topical field of pharmaceutical research. The general pharmacophoric model of an HDAC inhibitor can be outlined as follows: (*i*) A zinc binding group (ZBG) that coordinates the Zn^2+^ ion within the HDAC active site and that can be constituted by numerous chemical functions with chelating properties including t-FMK; (*ii*) a hydrophobic connection unit (LINKER) that accommodates the tubular access of the active site; (*iii*) a variable capping group (CAP) that interacts with the external domain of the enzyme and may discriminate different HDAC isoforms [126].

**Figure 17:**
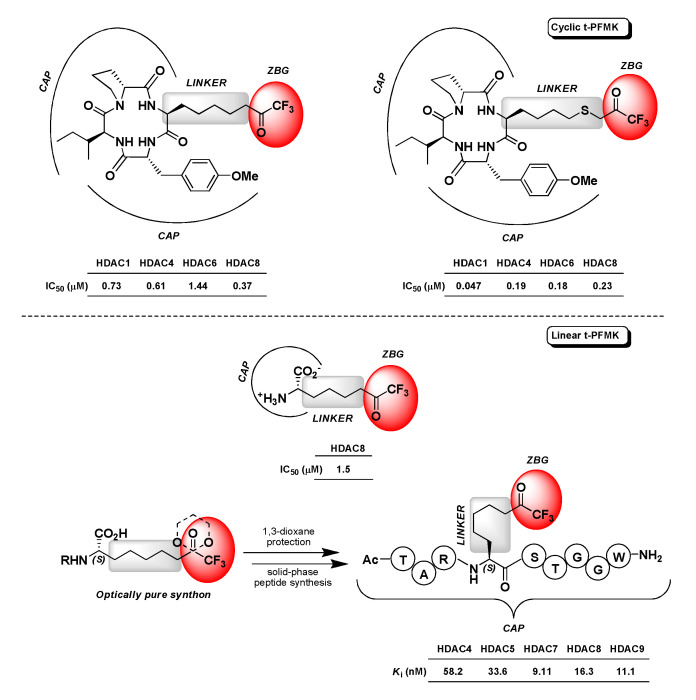
Structures and activity of the t-PFMKs developed as HDAC inhibitors.

Not as many t-PFMKs as HDAC inhibitors have been reported in the literature so far. Jose B. et al. developed two cyclic tetrapeptides (CAP) bearing a terminal t-FMK moiety (ZBG) connected through a poly(thio)methylene chain (LINKER) (Figure 17). These two t-PFMKs showed potent inhibition (low-micromolar to sub-micromolar range) towards four HDAC isoforms [127]. Another example in this regard is represented by (*S*)-2-amino-8,8,8-trifluoro-7-oxo-octanoic acid (Figure 17), which is actually an l-Arg analogue initially designed by Ilies M. et al. as a potential inhibitor of human arginase I that showed activity towards HDAC8 in the low-micromolar range (IC_50_ = 1.5 μM) [128]. More recently (2018), Moreno-Yruele and Olsen C.A. carried out a synthetic method to obtain a t-FMK-containing amino acid building block (specifically (*S*)-2-amino-8,8,8-trifluoro-7-oxo-octanoic acid, which corresponds to the LINKER-ZBG portion of a standard HDAC inhibitor) in optically pure form, and to exploit this synthon protected at the carbonyl of the t-FMK moiety (cyclic ketal) for the scalable, cost-effective, and automated solid-phase synthesis of various t-PFMKs. By means of this synthetic strategy they obtained a polypeptide-base t-FMK with nanomolar affinity towards class IIa HDACs (i.e., -4, -5, -7, and -9) and HDAC8 isoforms, whose simplified structure is reported in Figure 17 [129].

### 4.4. Viral Proteases Inhibitors

**Figure 18:**
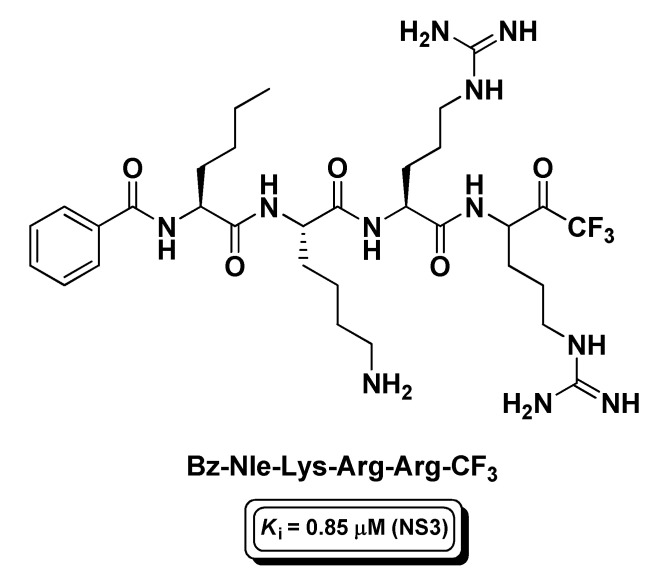
Structure and activity of the t-PFMK developed by Yin Z. et al. as Dengue virus NS3 inhibitor.

Another active research area pertaining to peptide-based t-FMKs is that of the development of protease inhibitors of viruses which are responsible for deadly human diseases. Among these enzymatic targets, one the most investigated has been the non-structural protease 3 (NS3) of Dengue virus, a mosquito-borne, (+)ssRNA virus of the *Flaviviridae* family that causes dengue fever. NS3 is a classical trypsin-like serine protease, whose catalytic activity depends on the interaction with the NS2B viral protein (co-factor) forming a complex (NS2B/NS3) which is essential for virus lifecycle [130]. However, unlike trypsin, NS3 shows a marked cleavage site preference for dibasic residues. On this premise, and on the basis of known non-prime side substrate preferences, Yin Z. and co-workers synthesized a tetrapeptidyl t-FMK, i.e., Bz-Nle-Lys-Arg-Arg-CF_3_ (Figure 18), with affinity for the intended target in the sub-micromolar range (*K*_i_ = 0.85 μM) [131]. This t-PFMK has been recently employed for molecular dynamics studies, from which it emerged that Bz-Nle-Lys-Arg-Arg-CF_3_ covalently binds the NS2B/NS3 complex via the Ser135 catalytic residue displaying a multi-binding mode behavior [132].

t-PFKMs have been also extensively studied as inhibitors of SARS-CoV 3CL^pro^, the above-described main protease of the coronavirus responsible for severe acute respiratory syndrome. A first set of derivatives containing Gln at the P_1_ site was provided by Sydnes M.O. et al. However, these compounds turned out to be poor inhibitors of this target [133]. Later on, Shao Y.-M. and co-workers synthesized a wider series of t-PFMKs, obtaining 3CL^pro^ inhibitors with IC_50_ values in the range 10–50 μM, with the tripeptidyl derivative Z-Ala-Val-Leu-CF_3_ showing the best activity (IC_50_ = 10 μM). This compound was then selected for kinetic studies by which was demonstrated a time-dependent inhibition profile, realistically due to the slow formation of the hemiketal adduct between the active site Cys145 residue and the carbonyl group of the FMK moiety [134].

**Figure 19:**
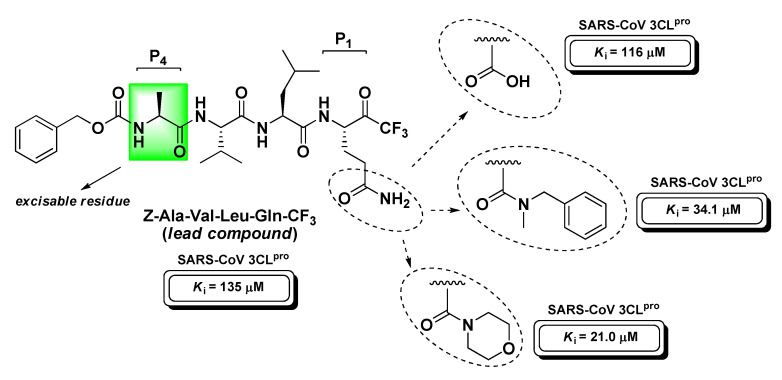
Chemical structure, SAR analysis, and activity against SARS-CoV 3CL^pro^ of the most relevant t-PFMK developed by Bacha U. et al.

Contextually, Bacha U. et al. developed a more complete series of peptide-based halomethyl ketones (including t-PFMKs) with the aim to further define the substrate preference of SARS-CoV 3CL^pro^ that can be summarized as follows: (*i*) Gln at P_1_ (essential requirement; possibly protected at the –NH_2_ group to prevent its nucleophilic attack to t-FMK carbonyl group avoiding cyclization and inactivation as previously described in the section of m-PFMKs); (*ii*) bulky aliphatic amino acids at P_2_ (Leu > Ile > Phe > Val > Met); (*iii*) P_3_ exposed to the solvent as is not believed to significantly affect the activity; (*iv*) small aliphatic amino acids at P_4_ (preferably Ala) [135]. The same research team continued their SAR investigations by varying the P_1_ site of the peptide backbone in order to impede the above-mentioned cyclization phenomenon [136]. Using Z-Ala-Val-Leu-Gln as a reference recognition motif for SARS-CoV M^pro^, they shortened the peptide sequence, eliminating the P_4_ site, and obtained three tripeptidyl t-FMKs with superior binding affinity, i.e., the morpholine derivative, the *N*-methyl-*N*-benzyl amide derivative, and the compound having Glu at the P_1_ site (Figure 19).

## 5. Conclusions

Although the development of PFMKs originated more than fifty years ago, in particular with the mono-fluorinated derivatives employed as selective activity-based probes for the study of druggable enzymatic targets, the enormous potential of this type of compound remains rather unexplored. d-PFMKs and t-PFMKs are gaining considerable attention within the pharmaceutical research area, as both di-fluorinated and tri-fluorinated methyl ketone moieties are now recognized as valid electrophilic pharmacophores for the design of therapeutic agents. Compared to m-PFMKs, d-PFMKs and t-PFMKs are more prone to undergoing nucleophilic attack at the *C*-terminal warhead (thus they may target a wide variety of hydrolytic enzymes such as serine and cysteine proteases), are able to impart superior physicochemical properties to the mere peptidic substrate (e.g., lipophilia and binding selectivity), and do not present particularly relevant metabolic drawbacks. The fluorinated motifs of these two functional groups, i.e., -CHF_2_ and -CF_3_, may also serve as bioisosteres (-CH_2_OH and -CH_2_CH_3_, respectively) to impart specific steric and electronic properties to a compound or to prevent its metabolic degradation. The entire electrophilic synthons, instead, are widely exploited as critical intermediates in organic synthesis of bioactive molecules. Furthermore, the ability of the di-fluorinated and tri-fluorinated methyl ketone moieties to exist in a stable hydrated form (thus mimicking the hydrolysis transition-state and acting as reversible competitive analogues) expands the field of application of the related PFMKs to other enzymatic targets (e.g., aspartic proteases). Therefore, consistent efforts are also currently directed to the stereoselective synthesis of these electrophilic moieties joined to a peptidic substrate with the aim of obtaining PFMKs in the optically pure form at the P_1_ site, another important requirement to increase the target-specificity. The growing interest and popularity of the d-FMK and t-FMK moieties relies also on the recent outcomes and future perspectives related to the development of compounds bearing such functional groups and their ability to tackle efficaciously a variety of viral infections and tumors (which are currently the major cause of death worldwide) by inhibiting specific enzymatic targets.

## Abbreviations

3CL^pro^	(Chymotrypsin-like protease)
Ac-PLVE-FMK	(Acetyl-Pro-Leu-Val-Glu(OMe)-CH_2_F)
Ac-PLVQ	(Acetyl-(Pro-Leu-Val-Gln))
Ac-VLPE-FMK	(Acetyl-Val-Leu-Pro-Glu(OMe)-CH_2_F)
AMPA	(α-amino-3-hydroxy-5-methyl-4-isoxazolepropionic acid)
Boc-Asp(OMe)-FMK	(Boc-Asp(OMe)-fluoromethyl ketone)
Casps	(Caspases)
Cat-B	(Cathepsin B)
Cat-C	(Cathepsin C)
Cats	(Cathepsins)
Cat-X	(Cathepsin X)
COVID-19	(Coronavirus disease 2019)
d-PFMKs	(Peptidyl di-fluoromethyl ketones)
DUBs	(Deubiquitinating enzymes)
EP1113	(Z-Val-(2-aminobenzoyl)-Asp-CH_2_F)
FP-2	(Falcipain-2)
HAV	(Hepatitis A virus)
HDAC	(Histone deacetylase)
HLE	(Human leukocyte elastase)
m-FMK	(mono-Fluoromethyl ketone)
m-PFMKs	(Peptidyl mono-fluoromethyl ketones)
M^Pro^	(SARS-CoV main protease)
Mu-Phe-HPhe-CH_2_F	(N-Morpholineurea-phenylalanyl-homophenylalanylfluoromethyl ketone)
NGLY1	(Peptide *N*-glycanase)
*N*-glycanase	(Peptide *N*-glycanase)
NK cells	(Natural Killer cells)
NMDA	(*N*-methyl-d-aspartic acid)
PFMKs	(Peptidyl fluoromethyl ketones)
PL^pro^	(Papain-like protease)
SARS-CoV	(Severe acute respiratory syndrome coronavirus)
SARS-CoV-2	(Severe acute respiratory syndrome coronavirus 2)
SENPs	(Sentrin/SUMO-specific proteases)
SUMOs	(Small ubiquitin-like modifiers)
TCP	(*P. falciparum* trophozoite cysteine proteinase)
THIQ-Leu-Phe-CH_2_F	(Tetrahydroisoquinoline Leu-Phe-CH_2_F)
TNFα	(Tumor necrosis factor)
t-PFMKs	(Peptidyl tri-fluoromethyl ketones)
ZBG	(Zinc binding group)
Z-DEVD-fmk	(Z-Asp(OMe)-Glu(OMe)-Val-Asp(OMe)-CH_2_F)
Z-DIPD-fmk	(Z-Asp(OMe)-Ile-Pro-Asp(OMe)-CH_2_F)
Z-IETD-fmk	(Z-Ile-Glu(OMe)-Thr-Asp(OMe)-CH_2_F)
Z-LEHD-fmk	(Z-Leu-Glu(OMe)-His-Asp(OMe)-CH_2_F)
Z-VAD-FMK	(Z-Valyl-alanyl-aspartyl-[*O*-methyl]-fluoromethyl ketone)
Z-VAD-fmk	(Z-Val-Ala-Asp-CH_2_F)
Z-YVAD-fmk	(Z-Tyr-Val-Ala-Asp(OMe)-CH_2_F)
β-CoVs	(β-Coronaviruses)
